# *Rhizophora mucronata* Lam. (Mangrove) Bark Extract Reduces Ethanol-Induced Liver Cell Death and Oxidative Stress in Swiss Albino Mice: In Vivo and In Silico Studies

**DOI:** 10.3390/metabo12111021

**Published:** 2022-10-25

**Authors:** Chitra Jairaman, Zeyad I. Alehaideb, Syed Ali Mohamed Yacoob, Sahar S. Alghamdi, Rasha S. Suliman, Anuradha Venkataraman, Bandar Alghanem, Senthilkumar Sivanesan, Rajagopalan Vijayaraghavan, Saranya Rameshbabu, Shree Mukilan Pari, Sabine Matou-Nasri

**Affiliations:** 1 PG & Research Department of Biotechnology, Mohamed Sathak College of Arts & Science, Shollinganallur, Chennai 600119, India; 2 Medical Research Core Facility and Platforms, King Abdullah International Medical Research Center (KAIMRC), King Saud bin Abdulaziz University for Health Sciences (KSAU-HS), Ministry of National Guard—Health Affairs (MNGHA), Riyadh 11481, Saudi Arabia; 3 Pharmaceutical Sciences Department, College of Pharmacy, KSAU-HS, MNGHA, Riyadh 11481, Saudi Arabia; 4 PG & Research Department of Biochemistry, Mohamed Sathak College of Arts & Science, Shollinganallur, Chennai 600119, India; 5 Department of Research and Development, Saveetha Institute of Medical and Technical Sciences (SIMATS), Thandalam, Chennai 602105, Tamil Nadu, India; 6 Department of Biosciences, Institute of Biotechnology, SIMATS, Thandalam, Chennai 602105, Tamil Nadu, India; 7 Molecular, Cell and Developmental Biology Department, University of California, Los Angeles, CA 48072, USA; 8 Cellular Therapy and Cancer Research Department, KAIMRC, KSAU-HS, MNGHA, Riyadh 11481, Saudi Arabia

**Keywords:** ethanol intoxication, liver injury, oxidative stress, necrosis, hepatoprotection, metabolites, *Rhizophora mucronata*

## Abstract

The bark extract of *Rhizophora mucronata* (BERM) was recently reported for its prominent in vitro protective effects against liver cell line toxicity caused by various toxicants, including ethanol. Here, we aimed to verify the in vivo hepatoprotective effects of BERM against ethanol intoxication with the prediction of potential targets employing in silico studies. An oral administration of different concentrations (100, 200 and 400 mg/kg body weight) of BERM before high-dose ethanol via intraperitoneal injection was performed in mice. On day 7, liver sections were dissected for histopathological examination. The ethanol intoxication caused liver injury and large areas of necrosis. The pre-BERM administration decreased the ethanol-induced liver damage marker tumor necrosis factor-alpha (*TNF-α*) expression, reduced hepatotoxicity revealed by nuclear deoxyribonucleic acid (DNA) fragmentation and decreased oxidative stress indicated by malondialdehyde and glutathione contents. Our in silico studies have identified BERM-derived metabolites exhibiting the highest predicted antioxidant and free radical scavenger activities. Molecular docking studies showed that most of the metabolites were predicted to be enzyme inhibitors such as carbonic anhydrase inhibitors, which were reported to stimulate the antioxidant defense system. The metabolites predominantly presented acceptable pharmacokinetics and safety profiles, suggesting them as promising new antioxidant agents. Altogether, the BERM extract exerts antioxidative activities and shows promising hepatoprotective effects against ethanol intoxication. Identification of related bioactive compounds will be of interest for future use at physiological concentrations in ethanol-intoxicated individuals.

## 1. Introduction

Ethanol, also called ethyl alcohol or alcohol, is considered a potent hepatotoxin capable of causing chronic liver damage [1]. Liver diseases, including alcoholic liver disorder (ALD), are associated with chronic alcohol abuse and have the highest morbidity and mortality globally [2,3]. The time and dosage contingent intake of alcohol increases the risk of ALD [4]. ALD progression is revealed by a series of liver diseases, beginning with fatty liver to swelling and noxious cells such as steatohepatitis, cholecystitis, cirrhosis, and finally, the development of hepatocellular carcinoma (HCC) [5].

Ethanol consumption and metabolism lead to high toxic levels of acetaldehyde by alcohol dehydrogenase, which generate oxidative stress [6]. Due to its highly reactive nature, acetaldehyde interacts with the cellular proteins, lipids, and DNA, causing adducts and the production of reactive species, which subsequently results in elevated hepatotoxicity and severe liver injury. In addition, the acetaldehydes mainly cause the formation of toxic and highly immunogenic protein adducts [7]. Consequently, acetaldehyde-adducted proteins and alcohol-induced oxidative stress increase the synthesis and release of tumor necrosis factor-alpha (TNF-α), an inflammatory cytokine, primarily secreted by the macrophages and demonstrated to contribute to liver injury and damage [8]. One of the factors with a major role in alcohol toxicity is the oxidative stress caused by the excessive generation of reactive oxygen species (ROS) [9]. Under normal physiological situations, the liver oxidative stress is regulated by the hepatic enzymatic (i.e., glutathione reductase) and non-enzymatic (i.e., reduced glutathione, GSH) antioxidant systems to maintain cellular redox homeostasis. The excessive alcohol consumption impairs the hepatic antioxidant system and leads to lipid peroxidation, indicated by malondialdehyde (MDA), and in GSH deficiency [10,11].

The consumption of ethanol leads to another major consequence, including cell fate and programmed cell death (i.e., apoptosis), a complex process characterized by deoxyribonucleic acid (DNA) fragmentation, which occurs in the liver [12] and in other tissues, including the brain [13], salivary gland [14] and gastric mucosa [15]. Alcohol toxicity also interferes with the electron transport chain that provokes mitochondrial dysfunction, apoptosis, cell damage, and ultimately necrosis, a form of premature cell death caused by autolysis and occurring in response to injury [16,17,18].

There is a growing interest in overcoming side effects caused by toxicants or conventional chemotherapeutic drugs leading to liver cell damage and hepatotoxicity resulting in apoptosis and necrosis, which are prominent in liver injury and liver diseases [19,20]. Cost-effective plant and plant-based preparations posing no side effects could be valuable for the treatment of liver disorders [20,21]. Based on a previous report, using human hepatocarcinoma cell line HepG2, the plant parts of *Rhizophora mucronata* Lam. (*R. mucronata*) (also known as Mangrove), including leaves, roots, flowers, bark and fruits, were shown to have promising therapeutic values for elephantiasis, hepatitis, ulcers through its antibacterial and anti-inflammatory activities, and the ability to neutralize toxicants, including ethanol intoxication [22,23]. However, no studies have explored the in vivo biological protective impact of the bark extract of *Rhizophora mucronata* (BERM) against ethanol-induced liver injury and hepatotoxicity. Thus, in this present study, BERM was tested for its potential hepatoprotective properties against ethanol intoxication in Swiss albino mice. This in vivo study was performed in an attempt to find a novel alternate and safe hepatoprotective drug against ethanol-induced liver injury. This study primarily focused on the assessment of the expression of liver injury biomarkers (i.e., *TNF-α* and cellular antioxidant *nuclear factor erythroid-2 related factor 2*, *NRF2*), on the evaluation of hepatotoxicity (i.e., apoptosis), and the measurements of oxidative stress-related components, MDA and the antioxidant GSH. The identification of BERM-derived metabolites with predicted antioxidant and free radical scavenger activities were estimated using an in silico approach. Hepatoprotective flavonoid milk thistle seeds-derived *Silybum marianum* (Silymarin), known for its antioxidative properties, was used as a positive control throughout this study.

## 2. Results

### 2.1. BERM Prevents Ethanol Intoxication-Induced Liver Necrosis and Injury

**Figure 1:**
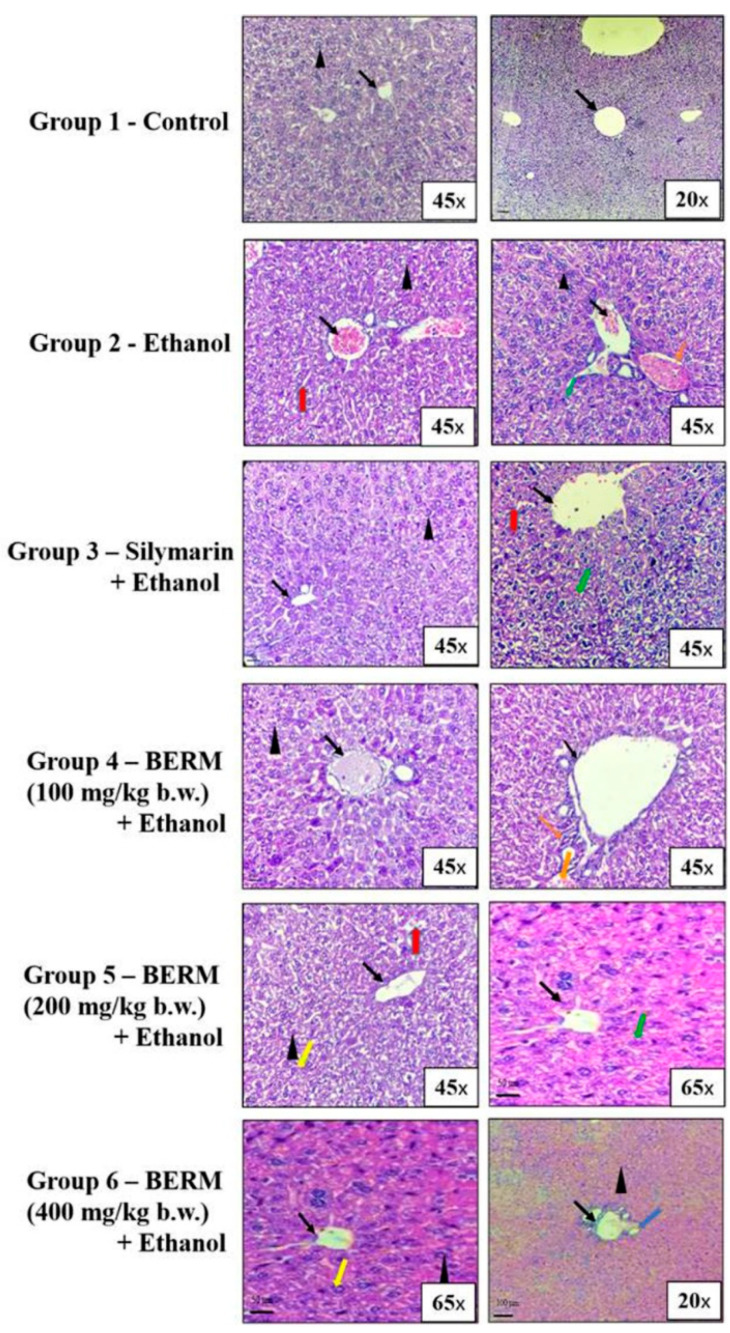
BERM pre-administration prevented ethanol intoxication-induced liver necrosis and injury in mice. Histopathological analysis of representative microsections of liver tissues extracted from the untreated (Group 1—Control) and treated mice (Group 2–6). From Group 2, high-dose ethanol-induced liver damage was observed in the tissues, as indicated by the arrows, pointing at damaged hepatic cells with microvacuolation (black arrowhead), granular cytoplasm (red arrow), and necrosis (orange arrow). Ethanol-induced liver injury was also observed, as indicated by the arrows, pointing at central vein damage (black arrow) and immune cell infiltration (green arrow). From Group 3, the oral pre-administration with Silymarin prevented ethanol-induced liver necrosis and injury as indicated by normal tissue architecture (normal central pointed by black arrow) and less damaged hepatic cells, granular cytoplasm, and cell infiltration. From Groups 4–6, the oral pre-administration with BERM (100–400 mg/kg b.w.) gradually prevented ethanol intoxication-induced liver damage as indicated by the absence of histopathologic alterations (no hepatic microvacuolations, pointed at by a black arrowhead) and areas showing recovery of the nucleus (pointed at by a yellow arrow) and normal hepatic artery (pointed at by the blue arrow), as compared to the Group 1 presenting no damage of the liver tissue, confirmed by normal hepatic cells (black arrowhead) and central vein (black arrow).

Based on the histopathological microsection examination of the liver tissues collected from the six groups of mice, different tissue characteristics were observed. In the group treated with high-dose ethanol alone, the liver tissue showed damaged hepatic cells, damaged central veins, vacuolar degeneration, necrosis and injury, a feature characterized by immune cell infiltration, compared to the normal untreated group (Figure 1). The groups of mice, which received treatment with Silymarin (the positive control) or BERM extract (100,200 and 400 mg/kg body weight, b.w.) before ethanol administration, showed a gradual improvement to normal tissue architecture as indicated by the absence of ethanol intoxication-induced necrosis and revealed normal central vein and nucleus recovery, compared to the control (Group 1), the untreated tissues (Figure 1). In addition, the oral pre-treatment with BERM followed by the administration of the toxic ethanol showed obvious prevention in toxic liver tissue injury confirmed by the absence of cell infiltration and hepatic microvacuolation, as compared to ethanol intoxication-induced liver injury (Figure 1).

### 2.2. BERM Reduces Ethanol-Induced TNF-α Gene Expression Level and Upregulates an Ethanol-Suppressed NFR2 Gene Expression Level

**Figure 2:**
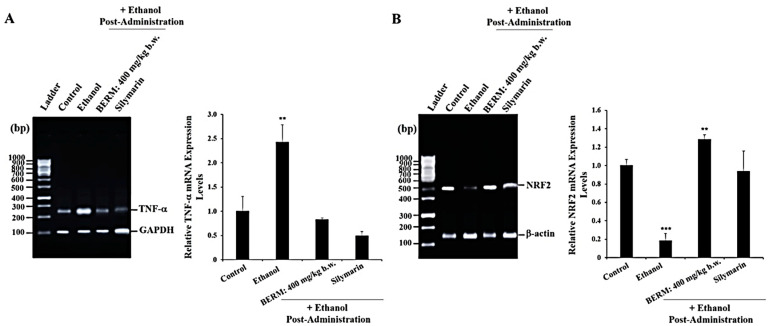
BERM pre-administration decreased ethanol-induced *TNF-α* mRNA expression levels and upregulated ethanol-suppressed *NRF2* mRNA expression levels. Representative gel electrophoresis showing mRNA expression levels of (**A**) TNF-α and the internal control glyceraldehyde 3-phosphate dehydrogenase (GAPDH), and (**B**) NRF2 and the internal control β-actin, determined by RT-PCR analysis in the control mouse group, ethanol-treated group, BERM, or Silymarin administration prior to ethanol injection. The bar graphs show the expression levels of *TNF-α* mRNA related to *GAPDH* and of *NRF2* related to *β-actin*. The data are presented as the mean ± standard deviation (SD) of three independent experiments. ** *p* < 0.01 and *** *p* < 0.001 vs. Control.

Chronic ethanol consumption also leads to the increase in the gene expression level of *TNF-α*, a pro-inflammatory cytokine used as a biomarker of liver injury [24]. As a biomarker of liver protection [25], the gene expression level of the antioxidant transcription factor *NRF2* was monitored. As measured by reverse transcriptase-polymerase chain reaction (RT-PCR) gel electrophoresis, the liver treatment with ethanol significantly upregulated the *TNF-α* messenger ribonucleic acid (mRNA) expression level (Figure 2A) and downregulated the *NRF2* mRNA expression level (Figure 2B) compared to the *TNF-α* and *NRF2* mRNA expression levels measured in the untreated control liver tissue (Figure 2). The pre-treatment with either BERM or Silymarin significantly decreased the ethanol-induced *TNF-α* mRNA expression level and upregulated the ethanol-suppressed *NRF2* mRNA expression level compared to the toxic high-dose ethanol-administered liver tissue (Figure 2).

### 2.3. BERM Reduces Ethanol-Induced Nuclear DNA Fragmentation

**Figure 3:**
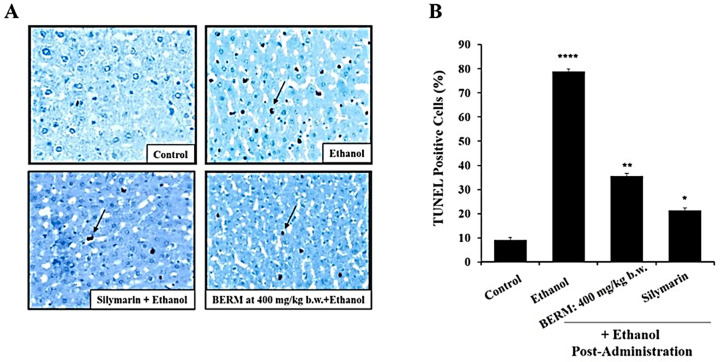
**BERM pre-administration decreased ethanol-induced programmed cell death.** (**A**) Representative photomicrographs showing apoptotic DNA fragments containing digoxigenin-labeled nucleotides were revealed using the TUNEL assay. The arrows point at examples of brownish apoptotic cells. (**B**) The bar shows the percentage TUNEL-positive apoptotic cells. The results are presented as the mean ± SEM based on three independent experiments. * *p* < 0.05, ** *p* < 0.01, and **** *p* < 0.0001 signify a statistically significant difference compared with the control.

Ethanol intoxication-induced liver injury leads to premature and programmed cell death, including apoptosis [26]. Known as a hallmark of apoptosis, nuclear DNA fragmentation was assessed using the terminal deoxynucleotidyl transferase (TdT) dUTP nick-end labelling (TUNEL) assay under a phase-contrast microscope. Mainly observed in the toxic alcoholic liver tissue, the shrunken cells with brown stained nuclei were identified as TUNEL-positive apoptotic cells, compared to the healthy cells that were observed in the untreated control liver tissue (Figure 3A). Fewer TUNEL-positive apoptotic cells were observed in the alcohol-induced liver injury after the pre-treatment with either BERM or Silymarin, as compared to the ethanolic liver tissue (Figure 3A). The apoptotic index was significantly decreased in ethanol intoxication-induced liver injury in the mice group pre-treated with BERM compared to mice treated with toxic ethanol alone (Figure 3B). The pre-treatment with Silymarin decreased the apoptotic index induced by ethanol intoxication at a higher level than the pre-treatment with BERM (Figure 3B).

### 2.4. BERM Lowers Ethanol-Induced MDA Levels and Increases Ethanol-Decreased GSH Levels in Liver Tissues

**Figure 4:**
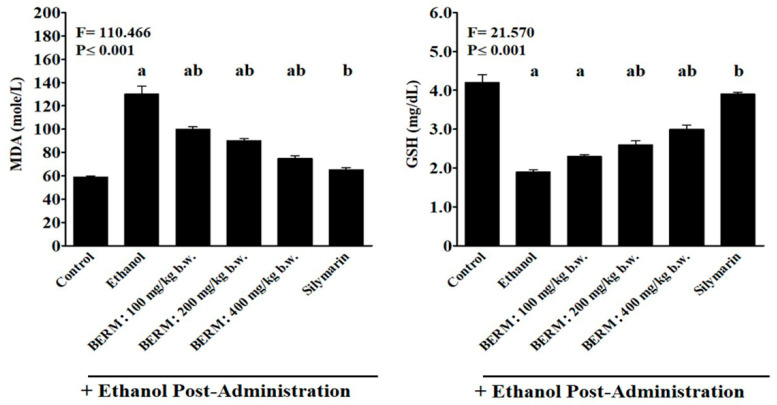
**Effect of BERM pre-administration on hepatic tissue GSH and MDA levels following to ethanol-induced liver damage.** The bar graphs show hepatic tissue levels of GSH and MDA measured using colorimetric methods involving specific substrates such as 5-5’-dithio-bis(2-nitrobenzoic acid) (DNTB) and 2-thiobarbituric acid (TBA) solutions, respectively. Refer to the methods section for more information. The letters a, b and ab, clearly indicate that they are statistically significant with the control (untreated) mouse group, with the ethanol group, and with the control and ethanol groups, respectively. The results are presented as the mean ± SEM based on five independent experiments.

As a biomarker of ethanol-induced oxidative stress and lipid peroxidation [11], the content of MDA was measured in the liver tissue homogenates. Ethanol administration significantly increased the level of MDA produced in liver tissue (Figure 4). The oral pre-treatment with Silymarin followed by the treatment with ethanol resulted in a significant decrease in ethanol-induced MDA production compared to the ethanol group (Figure 4). A significant gradual reduction in ethanol-induced MDA production was observed with increasing concentrations of oral pre-treatment with BERM extracts (100, 200 and 400 mg/kg b.w.) compared to the ethanol group (Figure 4). The decrease in ethanol-induced MDA production by the pre-treatment with BERM tested at 400 mg/kg b.w. was similar to the decrease in ethanol-induced MDA production in liver tissue caused by the pre-treatment with Silymarin (Figure 4).

The GSH levels were the lowest In the toxic ethanol group and the highest in the Silymarin group (Figure 4). A significant progressive increase in the ethanol-induced GSH production was observed by increasing concentrations of oral pre-treatment with BERM extracts (100, 200 and 400 mg/kg b.w.), compared to the ethanol group (Figure 4). A significant difference was observed between the increased level of GSH content detected in ethanol-induced liver damage pre-treated with BERM (400 mg/kg b.w.) and Silymarin groups (Figure 4).

### 2.5. Prediction of Antioxidant and Free Radical Scavenger Activities of BERM-Derived Metabolites Using Prediction of Activity Sprectra for Substances PASS Online Web Server

**Figure 5:**
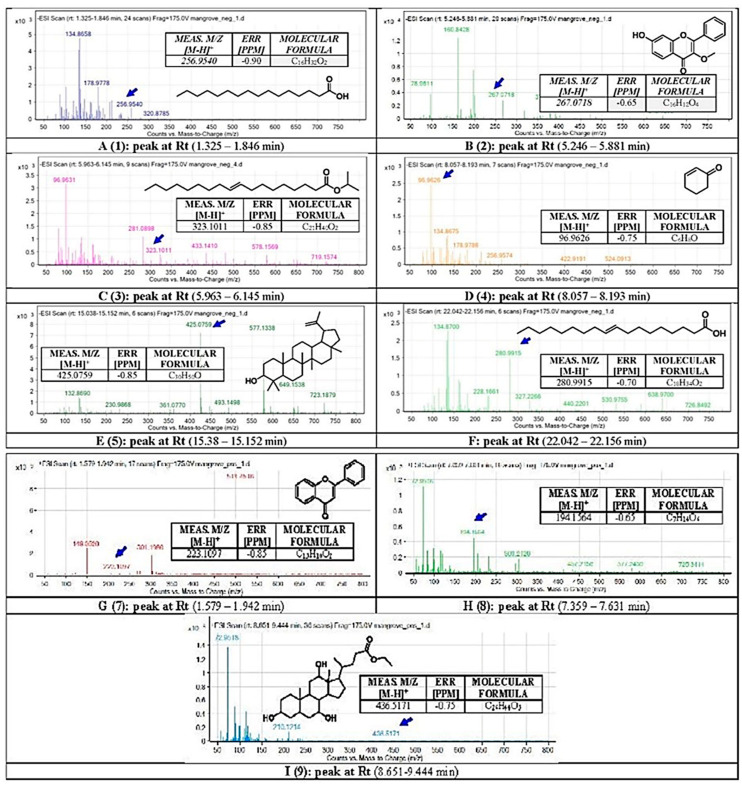
**Chemical analysis using Liquid Chromatography with Mass Spectrometry (LC-QTOF).** The secondary metabolites in BERM were tentatively identified as (**1**) n-Hexadecanoic acid, (**2**) 4H-1-benzopyran-4-one, 7-hydroxy-3-methoxy-2-pheyl, (**3**) Elaidic acid, isopropyl ester, (**4**) 2-Cyclohexen-1-one, (**5**) Lupeol, (**6**) Oleic acid, (**7**) Flavone, (**8**) O-methyl-d-glucose, and (**9**) Ethyl iso-allocholate. Means *m*/*z* implies measured *m*/*z*.

After presenting promising antioxidant activity in ethanol-intoxicated mice, nine BERM-derived metabolites were identified using the LCMS method: n-Hexadecanoic acid [27] referred to as Metabolite 1; 4H-1-Benzopyran-4-one, 7-Hydroxy-3-methoxy-2-phenyl [28] referred to as Metabolite 2, Elaidic acid, isopropyl ester [29] referred to as Metabolite 3, 2-Cyclohexen-1-one [27] referred to as Metabolite 4, Lupeol [28] referred to as Metabolite 5, Oleic acid [28] referred to as Metabolite 6, Flavone [28] referred to as Metabolite 7, 3-O-methyl-D-glucose [30] referred to as Metabolite 8, and Ethyl iso-cholate [30] referred to as Metabolite 9 (Figure 5). Therefore, the predicted antioxidant and free radical scavenger activities were determined for the identified metabolites using the PASS online web server. As shown in Table 1, Silymarin possessed the highest predicted antioxidant (0.859) and free radical scavenger (0.956) activities, followed by Metabolite **5** (0.594, and 0.743) and Metabolite **7** (0.469 and 0.469), respectively.

### 2.6. Molecular Target Predictions of BERM-Derived Metabolites Using the Molinspiration Web Server

The molecular targets for the identified BERM-derived metabolites were predicted and assessed using the Molinspiration cheminformatics web server to evaluate the biological targets that might be modulated by these metabolites. When the bioactivity score is greater than 0.00, it indicates that the molecule is active on this target, while a bioactivity score between −0.50 to 0.00 suggests an intermediate activity, and a score below −0.50 presumes that the molecule is inactive. The positive control Silymarin demonstrated the highest score as an enzyme inhibitor (0.23), and similar findings were observed with other metabolites, as summarized in Table 2.

### 2.7. Molecular Docking of BERM-Derived Metabolites into Carbonic Anhydrase (CA) II Enzyme

**Figure 6:**
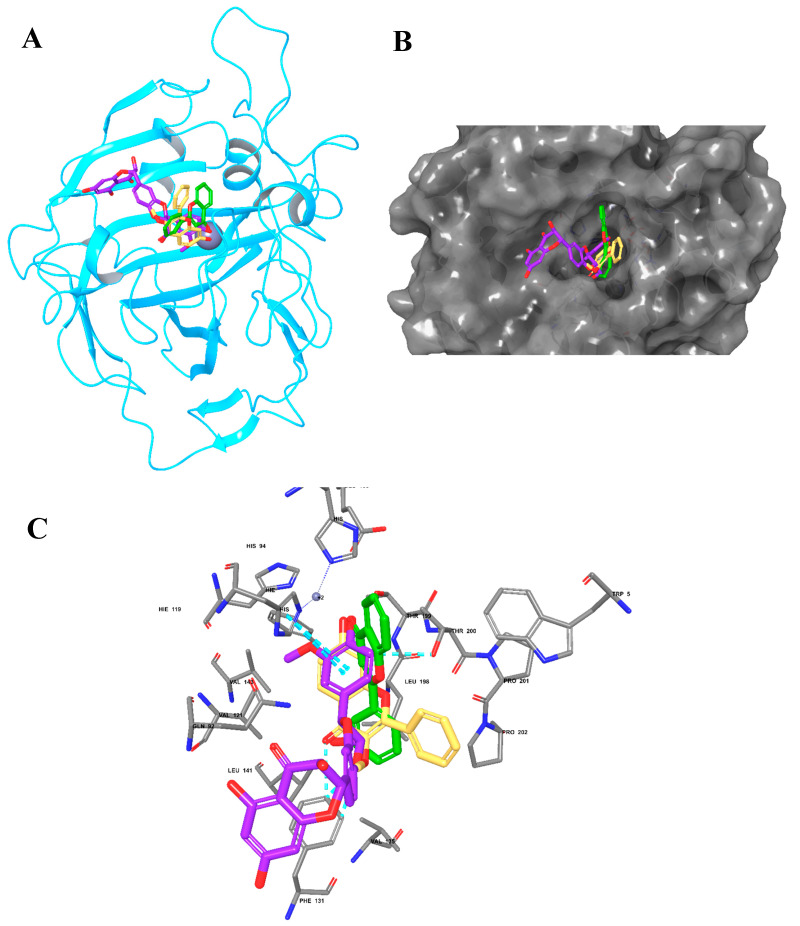
**The molecular docking of Silymarin and BERM-derived Metabolites into CA II Enzyme**. (**A**) Overlay of Silymarin (Violet), Metabolite 5 (faded orange) and Metabolite 7 (green) in the binding pocket of CA II enzyme; (**B**) surface representation of CA II enzyme with the docked Metabolites occupying the binding pocket; (**C**) the molecular interactions of Silymarin (Violet), Metabolite **5** (faded orange), and Metabolite **7** (green) with the amino acids in the binding site.

To assess potential binding interactions between the identified metabolites and the well-known antioxidant metalloenzyme CA II, a molecular docking study was performed using the Glide Maestro tool. Silymarin was shown to exhibit the highest docking score (−6.267) with several interactions, as summarized in Table 3, followed by Metabolites **5** and **7**. These results were consistent with bioactivity predictions in which Metabolites **5** and **7** demonstrated the highest predicted activity. Moreover, Metabolites **5** and **7** occupied a similar binding pocket as Silymarin, maintaining zinc coordination and amino acid interactions as shown in Figure 6.

### 2.8. Predictions of Absorption, Distribution, Metabolism and Excretion (ADME) Properties for Identified Bioactive BERM-Derived Metabolites

**Figure 7:**
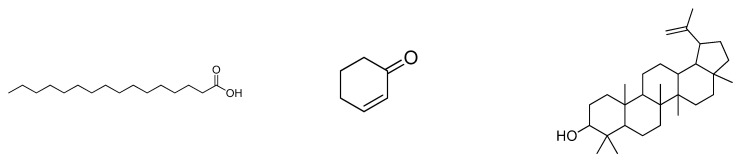

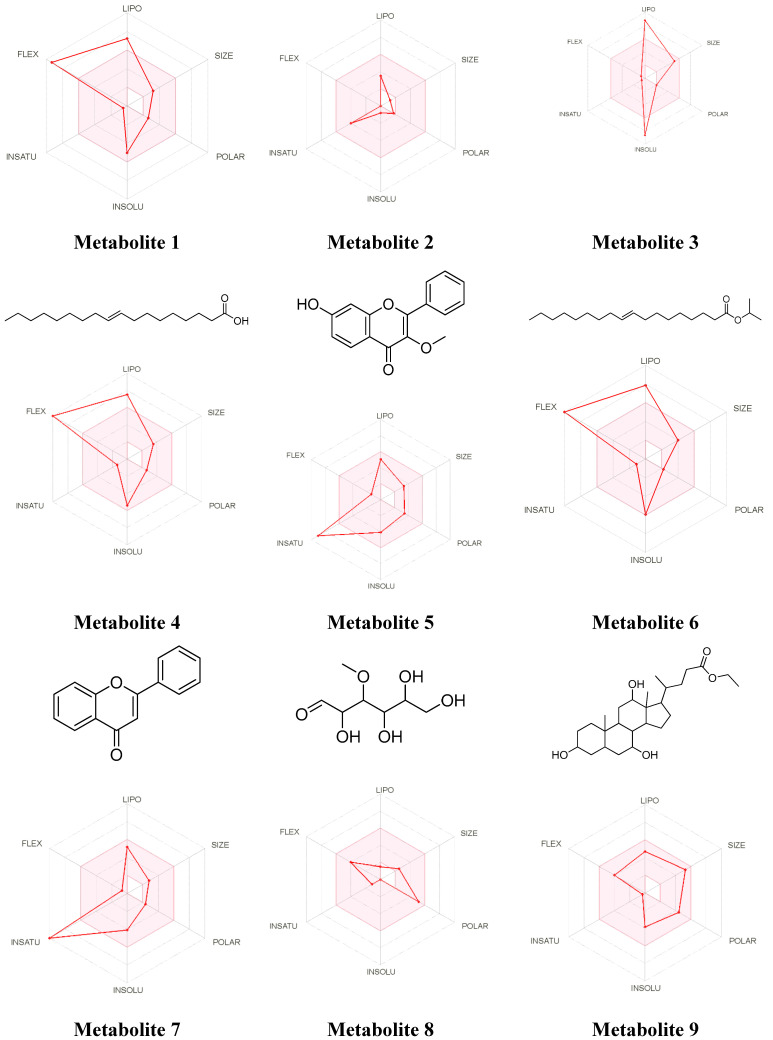
**The bioavailability radar for the identified bioactive Metabolites in BERM**. LIPO: Lipophilicity, Size: Molecular weight, POLAR: solubility, INSOLU: insolubility, INSATU: insaturation, and FLEX: flexibility. The properties within the colored zone are preferred for orally active drugs.

In order to investigate the physicochemical and pharmacokinetic properties of the identified metabolites, the SwissADME web server was used. Five important parameters were evaluated for Silymarin and identified metabolites, including molecular weight, lipophilicity (Log P), solubility (Log S), blood–brain barrier (BBB) penetration, and gastrointestinal (GI) absorption. All the metabolites were within the acceptable range according to Lipinski’s rule of five (ROF). However, the Metabolites **1**, **3**, **4** and **6** violated the ROF possessing high LogP values, as summarized in Table 4 and Figure 7.

### 2.9. Cytochrome P450 (CYP) Enzyme Inhibition Profile for the Identified Bioactive BERM-Derived Metabolites

After evaluation of their physiochemical properties, it was crucial to investigate the effects of the BERM-derived metabolites on CYP isoenzyme inhibition since it is a major mechanism responsible for drug–drug interactions. Thus, the BERM-derived metabolites were evaluated against several CYP isoenzymes, including CYP1A2, CYP2C19, CYP2C9, CYP2D6, and CYP3A4. Five out of nine metabolites were predicted to inhibit the CYP1A2 enzyme, while the other CYP types were less affected (Table 5), suggesting that fewer drug interactions are anticipated.

### 2.10. Organ Toxicity Predictions for the Identified Bioactive BERM-Derived Metabolites

The organ and endpoint toxicity of identified BERM-derived metabolites were predicted using the ProTox-II web server. As summarized in Table 6, none of the metabolites were predicted to cause hepatotoxicity, while only Metabolite **7** may possess a carcinogenic activity. Silymarin and Metabolite **9** were predicted to exhibit immunotoxicity. Moreover, Silymarin and the nine metabolites may lack mutagenicity and cytotoxicity, with the exception of Metabolite **7**, which was predicted to possess cytotoxic potential. With respect to the predicted toxicity class, all metabolites demonstrated class IV toxicity indicating less tendency to cause oral toxicity, while Metabolites **2** and **4** were below class IV.

## 3. Discussion

The liver is mainly involved in the detoxification of viral infection, prolonged drug therapy, various toxicants (i.e., carbon tetrachloride (CCl_4_) and environmental pollutants, industrial chemicals), and chronic alcoholism [19,31]. Metabolism and detoxification can generate numerous oxidative stress-related intermediate and end-products leading to hepatotoxicity, characterized by the hepatocyte death, liver damage, liver injury and eventually liver diseases [32]. These harmful free radicals and ROS can impair the prominent hepatic enzymatic and non-enzymatic antioxidant systems, decreasing the detoxification capacity of the liver [33]. Current conventional medical therapies for liver failure or liver diseases, such as drug-based treatment or even post-transplantation medication have side effects, mandating the urgent discovery of new plant and plant-based formulations as safe medication therapies [21]. In recent decades, the literature has reported the hepatoprotective activities of various natural products extracted from plants [34], including Silymarin reaching clinical trials [35]. We previously evaluated the in vitro hepatoprotective activities of BERM based on the reduction of cytotoxicity in the HepG_2_ cell line exposed to the combined BERM and toxicants (i.e., CCl_4_, ethanol and paracetamol) treatment [36]. In the current study, we demonstrated the hepatoprotective activities of BERM in ethanol-intoxicated mice. We found a concomitant reduction in ethanol-induced hepatotoxicity, revealed by downregulation of *TNF-α* gene expression, upregulation of *NRF2* gene expression, the induction of apoptosis, the decrease in the ethanol-induced MDA production, and an improvement in the ethanol-decreased GSH production. We also identified BERM-derived metabolites with predicted antioxidant activities, such as CA II inhibitors, presenting promising potentials as safe hepatoprotective plant-based drugs for individuals with ethanol intoxication.

As an important marker of liver damage, the *TNF-α* gene expression level was monitored in liver tissue homogenates. In this present study, the prolonged exposure to ethanol resulted in an increased level of the *TNF-α* gene in the toxic study group. Similar results were obtained by Nowak and Relja [37] who demonstrated that the NF-κB signaling pathway was activated during alcoholic liver disease, resulting in the stimulation of pro-inflammatory cytokine and chemokine gene expression. In humans, chronic alcohol consumption is associated with increased production of serum pro-inflammatory cytokines (e.g., TNF-α, interleukin (IL)-1, IL-6, IL-8) [38,39]. The correlation between the inflammation and oxidative stress during alcoholic liver injury is indisputable. Improper metabolism of ROS results in the expression of hypoxia-inducible factor-1 alpha that may also increase TNF-α secretion, prompting an immune reaction that intensifies the liver damage [40]. In addition to triggering inflammation, the TNF-α bound to its receptor initiates the programmed cell death pathways, such as apoptosis through the activation of downstream kinases and proteases, including caspases [41]. A deeper investigation of the reverse effect of ethanol intoxication inducing apoptosis-related molecular mechanisms, including caspase-dependent (extrinsic) and mitochondria-dependent (intrinsic) pathways, contributing to the hepatoprotective activities of BERM, would be of interest.

The TUNEL assay was applied to detect apoptotic cells that undergo massive DNA fragmentation during the final stages of apoptosis. DNA damage may be incurred due to exposure of the hepatocytes to ethanol intoxication-induced oxidative stress, causing the production of ROS and TNF-α-induced cell death, resulting in hepatic damage [26]. The current study showed that the ethanol intoxication of mouse hepatocytes increased the number of apoptotic cells observed with the TUNEL assay and phase-contrast microscopy, which is consistent with the literature using human alcoholic hepatitis specimens [42,43]. However, in the current study, when pre-treated with either Silymarin or BERM, a substantial decrease in the number of ethanol-induced apoptotic cells was noticed, confirming the in vivo hepatoprotective effect of BERM against alcohol intoxication. The recently reported purification and isolation of unidentified bioactive compounds from BERM along with those characterized BERM-derived hepatoprotective agents, including daidzein, epicatechin, hesperidin, diosmin, and quercetin [30,44], will pave the way for the development of alternative sources of safe and promising novel hepatoprotective agents against toxic liver disorders.

Oxidative stress is associated with the pathological process of ethanol-induced liver damage [45] and is regulated by the cellular antioxidant transcription factor NRF2, a master regulator of lipid metabolism [25]. In the current study, the administration of BERM upregulated ethanol-suppressed *NRF2* gene expression. Using a specific NRF2 knockout mouse model, a recent study demonstrated the critical role of the hepatic NRF2-mediated ethanol detoxification responses in preventing the development of ALD and ethanol toxicity-induced liver injury [25]. The production of MDA, known as an oxidative stress marker and as a marker of lipid peroxidation, was substantially enhanced in the ethanol-treated group compared to the untreated control group. The MDA overproduction due to ethanol-induced liver damage is aligned with the literature [46]. Another study reported that oxidative stress in the brain due to ethanol consumption also elevated MDA levels [47]. GSH is a crucial non-enzymatic antioxidant related to oxidative stress, which scavenges hydrogen peroxide (H_2_O_2_) radicals and reacts directly with certain ROS (e.g., the hydroxyl radical) and reverses its toxic effects. In the present study, the ethanol toxicity group exhibited reduced levels of GSH compared to the control and treated groups, resulting in the reduced synthesis of GSH, as previously reported [48]. Observed in rats subjected to alcohol and tobacco smoke exposure, the generation of oxidative stress also decreased the GSH levels in the liver [49], similar to the current findings.

The identified BERM-derived metabolites were characterized using in silico approaches. The antioxidant and free radical activity predictions suggest that Metabolites **5** and **7** may possess experimental activity compared to the positive control Silymarin. Moreover, molecular target prediction demonstrated high scores for the metabolites as enzyme inhibitors, which were also consistent with Silymarin-related scores. The obtained predictions propose that metabolites could share a similar enzyme target similar to that of Silymarin. Since Silymarin was previously reported to inhibit the CA II enzyme [50,51], we investigated the binding mode and molecular interactions with identified metabolites and the CA II enzyme. Of note, CA II inhibitors have been reported to reduce oxidative stress through the activation of the first-line antioxidant defense enzymes, including catalase and superoxide dismutase [52,53]. Additionally, several studies have demonstrated that phenolic and flavonoids, the largest phytochemical compounds in plants, possess an inhibitory activity on CA II enzymes [54,55]. Our results showed that Metabolites **5** and **7** exhibited the highest docking scores and comparable binding modes similar to Silymarin. Moreover, the pharmacokinetic properties are an essential part of drug discovery and development [56], and most identified metabolites were within the acceptable range of ROF, except for a few metabolites that violated the lipophilicity rule. The safety aspects of the identified metabolites demonstrated that CYP1A2 could be inhibited, while the effect on other CYP isoenzymes was minimal. In addition, most of the metabolites were classified as class IV indicating moderate oral toxicity predictions.

## 4. Materials and Methods

### 4.1. Reagents

All reagents and consumables were purchased from Sigma-Aldrich Corp. (St. Louis, MO, USA) unless mentioned otherwise.

### 4.2. Sample Collection and Extract Preparation

In January 2018, the barks of *Rhizophora (R.) mucronata* Lam. were found in Pichavaram Mangrove forest (latitude: 11°23′ to 11°30′ N; longitude: 79°45′ to 79° to 50′ E) and authenticated by Jayaraman with specimen No: PARC/2018/3854 at the Herbal Plant Anatomy Research Centre, West Tambaram, Chennai, India. The bark was dried in the shade for 15 days, coarsely powdered and kept in airtight containers, then used for research.

The pre-weighed 500 g of powdered bark of *R. mucronata* were brought in an airtight glass container and soaked in ethanol: water (3:1) weighing about 1500 mL. The container was sealed and kept for 2 weeks with sporadic mixing and agitation. The crude bark extract of *R. mucronata* (BERM) was filtered through a Grad I Whatman^®^ filter paper and evaporated at an ambient temperature and refrigerated at 4 °C for further use.

### 4.3. Animal Procurement and Maintenance

The animals were procured from Biogen Laboratory Animal Facility (Bangalore, Karnataka, India). For the present study, the healthy male Swiss C57/BL/6 Albino mouse strains (*n* = 36) were from 8 to 10 weeks and weighed 25 to 30 g. The animal-based experiments were performed in accordance with the ethical norms and guidelines of the Committee for the Purpose of Control and Supervision of Experiments on Animals (CPCSEA, New Delhi, India) and approved by the Institutional Animal Ethical Committee (IAEC) of Saveetha Medical College (SU/CLAR/RD/002/2018).

The mice were transferred to laboratory conditions 10 days before the start of the experiment for acclimatization. The mice were kept in plastic cages and were marked on the tail to identify each individual. Throughout the experiment, the mice were fed with ADILAID^®^ Hemster vegetable pellets (Mubai, India; Supplementary file S1) and drank potable water ad libitum, except during the short 2 h fasting period before the treatment when the food supply was still ad libitum but without the drinking water.

### 4.4. Animal Study Design and Sample Preparation

The experiment was designed according to the published protocol [30]. The 36 Swiss Albino mice were divided into 6 groups. **Group 1**: Standard control group. For 6 days, the mice received (5 mL/kg body weight, b.w.) distilled water orally (i.e., *per os*). **Group 2**: Ethanol-induced liver injury group. Only high-dose ethanol (cat. #64-17-1, Sigma-Aldrich Corp., St. Louis, MO, USA) was administered to the mice. **Group 3**: Silymarin (cat. #S0292, Sigma-Aldrich Corp.) + ethanol group. A single dose of Silymarin (50 mg/kg b.w.) was administered *per os* prior to the ethanol administration. **Group 4**: 100 BERM + ethanol group. A single dose of 100 mg/kg b.w. BERM was administered *per os* prior to the ethanol administration. **Group 5**: 200 BERM + ethanol group. A single dose of 200 mg/kg b.w. BERM was administered *per os* prior to the ethanol administration. **Group 6**: 400 BERM + ethanol group. A single dose of 400 mg/kg b.w. BERM was administered *per os* prior to the ethanol administration. Except for the untreated mice in Group 1, all the treated mice were given ethanol (5 mL/kg b.w. of 25% *w*/*v* ethanol) via intraperitoneal (i.p.) injection for 6 days, half an hour after oral administration of the plant extract.

On day 7, the mice were anesthetized by isoflurane inhalation followed by cervical dislocation [57]. The liver was removed, thoroughly rinsed with regular brine and dried with tissue paper. The left upper lobe of the liver was cut with sterile scissors and covered with aluminum foil, and stored at −70 °C before processing RT-PCR for the *TNF-α* and *NRF2* gene expression level monitoring and TUNEL assays. The remaining part of the liver (approximately 10%) was homogenized using a tissue homogenizer (MC Dalal & Co, Chennai, India). The homogenized tissue was prepared in a phosphate buffer (0.2 M, pH 7.4). After centrifugation at 2075× *g* for 15 min, the tissue homogenate was utilized for the detection of MDA and GSH levels.

### 4.5. Histopathological Analysis

The liver was first fixed in 10% formalin, followed by a dehydration process using concentrations of ethanol (50–100%). Then, the tissue was rinsed with xylene and impregnated in paraffin wax. The liver tissue sections (5–6 μm thickness) were generated using a rotary microtome and later stained with haematoxylin and eosin (H&E) dye for histopathological examination.

### 4.6. RNA Extraction and RT-PCR

Total RNA was isolated with ONE STEP-RNA Reagent (Biobasic Inc., Amherst, NY, USA) from the untreated and treated liver tissue homogenates. The concentration and quality of RNA samples were assessed using ultra-violet spectrophotometry (Tinzyme, New Delhi, India). Easy Script Plus™ Reverse Transcriptase (Lamda Biotech., St. Louis, MO, USA) was used for the reverse transcription of high-quality RNA extracts. Briefly, 0.5 µg total RNA, 2 µL oligo dT and 0.5 µg/mL random hexamer primers in diethyl pyrocarbonate (DEPC)-treated water were inoculated for 5 min at 65 °C and instantly cooled on ice. After the addition of 4 µL dithiothreitol (10 mM), 2 µL dNTP (10 mM) and 8 µL First Strand buffer, the temperature of the solution was lowered to 55 °C and completed with 200 U Easy Script Plus™ Reverse Transcriptase. The solution was then incubated at 55 °C for 60 min, then at 85 °C for 15 min to generate complementary DNA (cDNA). The *TNF-α*, *NRF2*, and internal controls *GAPDH* and *β-actin* genes were amplified by PCR using selected primer pair sequences as follows: *TNF-a* (GenBank accession No. Y00467), 5′-ACCCTCACACTCAGATCATCTTA-3′ (*forward*), 5′-TGGTGGTTTGCTACGACGT-3′ (*reverse*); *NRF2* (GenBank accession No. U20532), 5′-ATCGACAGTGCTCCTATGCGTGAA-3′ (*forward*), 5′-ATCGTCTGGGCGGCGACTTTAT-3′ (*reverse*); *GAPDH* (GenBank accession No. GU214026)*,* 5′-GCAAGTTCAACGGCACAGTCAAG-3′ (*forward*), 5′-ACATACTCAGCACCAGCATCACC-3′ (*reverse*); *β-actin* (GenBank accession No. NM007393)*,* 5′-GGGACCTGACTGACTACCTCA-3′ (*forward*), 5′-GACTCGTCATACTCCTGCTTG-3′ (*reverse*). The PCRs were performed in duplicate for each sample. Pfaffl’s mathematical model was used to calculate the relative quantification of *TNF-α* and *NRF2* transcripts [58]. In a total volume of 25 mL, 1.5% agarose and 1X Tris-acetate-EDTA (TAE) buffer were prepared and cascaded onto a gel tray. The loading dye was blended with the PCR product. With the 1 kilobase pair (kbp) DNA ladder used as a reference, sample mixes were loaded into each well. The gel was run at 50 V for 90 min; then, the PCR products were visualized with ethidium bromide staining. The Gel Pro Analyzer software (version 4.0, Roper Technologies, Inc., Sarasota, FL, USA) was used for the transcript quantification analysis. The quantity of the *TNF-α* and *NRF2* transcripts were related to *GAPDH* and *β-actin* transcripts, respectively.

### 4.7. TUNEL Assay

DNA fragmentation analyses were carried out in the paraffin-embedded liver tissue using a terminal deoxynucleotidyl TUNEL reaction according to the manufacturer’s instructions. The TUNEL reaction mixture (250 μL) was formulated using: TdT (25 μL) diluted in the nucleotide mixture (225 μL). The nucleotide solution without the TdT was the negative control in all experiments. After cell lysis and DNA strand decondensation, the slides containing the paraffin-impregnated liver tissue were washed twice with PBS. A drop of the TUNEL reaction mix (25 μL) was placed on each slide and a coverslip was added for mounting. The slides were incubated in a dark and highly moist chamber for 60 min at 37 °C. The coverslips were removed and the slides were washed three times with PBS. The slides were developed with diaminobenzidine substrate, counterstained with H&E dye, and scrutinized for the confirmation of apoptosis, as revealed by DNA fragmentation. The count of brown apoptotic cells was normalized to the total cell count as visualized by H&E staining. The apoptotic index was calculated by dividing the number of apoptotic cells by the total number of cells in random fields.

### 4.8. Oxidative Stress-Related Biochemical Assays

#### 4.8.1. Estimation of Reduced Glutathione

The GSH was measured in the liver tissue homogenate as described in [59]. Briefly, to precipitate the proteins, 125 μL of 25% of trichloroacetic acid (TCA) were added to 0.5 mL of tissue homogenate. The test tubes were chilled on ice for 5 min and the supernatant was diluted with 0.6 mL of 5% TCA and centrifuged for 10 min at 9000× *g*. A volume of 0.7 mL of sodium phosphate buffer (0.2 M, pH 8.0) was added to the aliquot (0.3 mL), to increase the mixed solution to 1 mL, and 2.0 mL of 5-5′-dithio-bis(2-nitrobenzoic acid) (DTNB) solution was added. After 10 min, the absorbance of the yellow color produced by the presence of 2-nitro-5-thiobenzoic acid, generated from the reduced glutathione GSH and DTNB reaction was measured using a spectrophotometer at a wavelength of 412 nm (Lovibond, ACD Company, New Delhi, India). Similarly, standards were included to measure the level of the GSH.

#### 4.8.2. Estimation of Malondialdehyde

The measurement of MDA detected in the liver tissue homogenate was completed as described in [60]. Briefly, 0.03 M Tris-HCl buffer (pH 7.4) and 0.2 mM sodium pyrophosphate were added to 0.2 mL of tissue homogenate for a total volume of 2 mL. The mixed solution was incubated at 37 °C for 20 min. The biochemical reaction was terminated with the addition of 1 mL of 10% TCA, after which 1.5 mL of the organic compound 2-thiobarbituric acid (TBA) was added, and the solution was heated. The resulting pink-colored product revealing the presence of MDA due to fatty acid oxidation was measured using the Lovibond^®^ spectrophotometer at an absorption of 535 nm.

### 4.9. Identification of BERM Metabolites Using LC-Q-TOF

The identification of BERM metabolites was performed using the Agilent 1260 Infinity high-performance liquid chromatography system (Agilent Technologies, Santa Clara, CA, USA) coupled to Agilent 6530 Quadrupole Time of Flight (Q-TOF). The analysis was performed using an Agilent SB-C18 column (4.6 mm × 150 mm, 1.8 μm) with the following elution gradient; 0–2 min, 5% B; 2–17 min, 5–100% B; 17–21 min, 95% B; 21–25 min, 5% B, using mobile phase A (0.1% formic acid in water) and mobile phase B (0.1% formic acid in methanol). The flow rate was set at 250 µL/min, and the injection volume was 10 µL. The scanning range was set at 50–800 (*m*/*z*), and the remaining parameters were set as follows: the gas temperature at 300 °C, gas flow of 8 L/min, nebulizer pressure of 35 psi, sheath gas temperature at 350 °C, and sheath gas flow rate of 11 L/min. The high-resolution masses were measured using the Agilent MassHunter qualitative analysis software (version B.06.00).

### 4.10. Prediction of Antioxidant and Free Radical Scavenger Activity

The antioxidant and free radical scavenger activity of the identified metabolites were predicted using the PASS online web server [61]. The web server predicts activity based on in-house and commercially available databases. It gives two main probabilities based on the training set; one is for active (P_a_) and the other for the inactive (P_i_). The higher predicted P_a_ value suggests that the compound might be experimentally active (http://way2drug.com/passonline/index.php, accessed on 21 March 2022).

### 4.11. Prediction of Molecular Target

The molecular targets for the identified metabolites were predicted using a Molinspiration (Web-enabled software for large-scale calculation of molecular properties and database searches, Free online molecular descriptor calculations, 2020) web-based tool (https://www.molinspiration.com/, accessed on 21 March 2022). The metabolite structure was utilized as input to generate the predicted molecular targets based on a database with known actives. The higher the score, the more likely the molecule is active at this target.

### 4.12. Molecular Docking into CA II Enzyme

The molecular docking of the identified metabolites and of the CA II enzyme was studied using Glide Schrödinger software (release 2022-2). The 2D chemical structures of the metabolites were prepared using LigPrep tool (Schrödinger Release 2021-4: LigPrep, Schrödinger, LLC, New York, NY, USA, 2021). The PDB file of CA II enzyme (5LJQ, Resolution: 1.05 Å) was downloaded from the Protein Data Bank (https://www.rcsb.org/, accessed on 20 March 2022) and prepared using the Protein Preparation Wizard tool. The active site grid in the enzyme was generated using the Receptor Grid Generation tool, and metabolites were docked using Glide docking in Schrödinger software (Schrödinger Release 2021-4).

### 4.13. Predictions of ADME Properties

The ADME properties of the identified metabolites were studied using the SwissADME webserver (http://www.swissadme.ch/, accessed on 20 March 2022). The chemical structures were used as input to generate ADME and drug-likeness properties [62]. In this study, we calculated the molecular weight, lipophilicity (Log P), solubility (Log S), blood–brain barrier (BBB) penetration, oral absorption, and violation of Lipinski’s rule of five (ROF).

### 4.14. CYP Enzyme Inhibition Profiling for the Identified Bioactive Metabolites

The effects of the identified metabolites on the inhibition of CYP enzyme were predicted using the SwissADME web server. The web server outputs are based on a training and testing set that could generate a prediction for a given chemical structure. Predicted inhibition includes several CYP isoenzymes such as CYP1A2, CYP2C19, CYP2C9, CYP2D6, and CYP3A4 [63].

### 4.15. Organ Toxicity and Safety Predictions

To predict the toxicity of identified metabolites, the ProTox-II website was utilized to generate organ and endpoint toxicity predictions [63]. The chemical structures were used as input data and various toxicity endpoints were evaluated such as hepatotoxicity, cytotoxicity, carcinogenicity, mutagenicity, and immunotoxicity (https://tox-new.charite.de/protox_II/, accessed on 20 March 2022).

### 4.16. Statistical Analysis

Sigma Plot-13 software (version 14) was used to carry out statistical analysis. The results are expressed as the mean ± standard deviation (SD) or the mean ± standard error of the mean (SEM). One-way ANOVA with Student–Newman–Keuls method for multiple comparisons was used to assess the significance of difference. If *p* < 0.05, the data were considered statistically significant.

## 5. Conclusions

The discovery of new plant-based antioxidant bioactive compounds capable of reversing the deleterious effects of toxicants, including ethanol intoxication, is of considerable interest. In this present study, BERM crude extract at the concentration of 400 mg/kg b.w. showed the highest protective effect against ethanol intoxication-induced liver damage in mice, comparable to the standard hepatoprotective herbal drug, Silymarin. The protective effect of BERM was accompanied by a decrease in a hepatic *TNF-α* gene expression, apoptotic nuclear DNA fragmentation and antioxidant MDA levels, as well as an increase in hepatic *NRF2* gene expression and antioxidant GSH levels. In addition, our in silico study revealed that some of the identified BERM-derived metabolites might possess promising antioxidant and free radical scavenger activity and were predicted to act as CA II inhibitors, which would exhibit antioxidant properties. As the computed predictions are insufficient, further fractionation and isolation of BERM-derived metabolites are needed to study the molecular mechanisms underlying the hepatoprotective effect of BERM against ethanol intoxication and explore the antioxidant effects of these metabolites through hepatic CA II enzymatic activity. Additional in vivo studies are also requested for further discovery and development of newly identified antioxidant hepatoprotective BERM-derived agents against ethanol intoxication-induced liver damage.

## Figures and Tables

**Table 1:** Bioactivity scores for antioxidant and free radical scavenger activities of identified BERM-derived metabolites using the PASS online web server.

Compound/ Metabolite (Antioxidant Activity)	Probability of Being Active (P_a_)	Probability of Being Inactive (P_i_)
**Silymarin**	0.859	0.003
**1**	0.222	0.045
**2**	0.147	0.106
**3**	0.280	0.027
**4**	0.283	0.026
**5**	0.594	0.005
**6**	0.278	0.028
**7**	0.469	0.008
**8**	-	-
**9**	0.181	0.068
**Compound/** **Metabolite** **(Free radical scavenger)**	**(P_a_)**	**(P_i_)**
**Silymarin**	0.956	0.001
**1**	0.315	0.027
**2**	0.201	0.075
**3**	-	-
**4**	0.360	0.021
**5**	0.743	0.003
**6**	0.357	0.022
**7**	0.469	0.012
**8**	-	-
**9**	-	-

**Table 2:** Predicted biological targets for BERM-derived metabolites using the Molinspiration web server.

Compound/ Metabolite	Molinspiration Scores	
**Silymarin**	G protein-coupled receptor (GPCR) ligand	0.07
	Ion channel modulator	−0.05
	Kinase inhibitor	0.01
	Nuclear receptor ligand	0.16
	Protease inhibitor	0.02
	Enzyme inhibitor	0.23
**1**	GPCR ligand	0.02
	Ion channel modulator	0.06
	Kinase inhibitor	−0.33
	Nuclear receptor ligand	0.08
	Protease inhibitor	−0.04
	Enzyme inhibitor	0.18
**2**	GPCR ligand	−3.51
	Ion channel modulator	−3.34
	Kinase inhibitor	−3.84
	Nuclear receptor ligand	−2.89
	Protease inhibitor	−3.43
	Enzyme inhibitor	−2.82
**3**	GPCR ligand	0.27
	Ion channel modulator	0.11
	Kinase inhibitor	−0.42
	Nuclear receptor ligand	0.85
	Protease inhibitor	0.15
	Enzyme inhibitor	0.52
**4**	GPCR ligand	0.17
	Ion channel modulator	0.07
	Kinase inhibitor	−0.22
	Nuclear receptor ligand	0.23
	Protease inhibitor	0.07
	Enzyme inhibitor	0.27
**5**	GPCR ligand	−0.21
	Ion channel modulator	−0.30
	Kinase inhibitor	−0.01
	Nuclear receptor ligand	−0.01
	Protease inhibitor	−0.47
	Enzyme inhibitor	0.11
**6**	GPCR ligand	0.05
	Ion channel modulator	−0.04
	Kinase inhibitor	−0.24
	Nuclear receptor ligand	0.14
	Protease inhibitor	0.02
	Enzyme inhibitor	0.12
**7**	GPCR ligand	−0.30
	Ion channel modulator	−0.21
	Kinase inhibitor	−0.12
	Nuclear receptor ligand	−0.18
	Protease inhibitor	−0.52
	Enzyme inhibitor	0.03
**8**	GPCR ligand	−0.63
	Ion channel modulator	−0.15
	Kinase inhibitor	−0.85
	Nuclear receptor ligand	−0.66
	Protease inhibitor	−0.35
	Enzyme inhibitor	0.20
**9**	GPCR ligand	0.17
	Ion channel modulator	0.21
	Kinase inhibitor	−0.38
	Nuclear receptor ligand	0.65
	Protease inhibitor	0.18
	Enzyme inhibitor	0.58

**Table 3:** SP and XP docking scores for identified BERM-derived metabolites.

Compound/Metabolite	Docking Score	Interactions with Amino Acid Residues
**Silymarin**	−6.267	Asp72, Glu 69, Asn 67, Ile 91, Gln 92, His 94, Phe 131, and Zinc coordination
**1**	-	-
**2**	−4.381	Zinc coordination
**3**	−2.857	-
**4**	-	-
**5**	−5.520	His94, Thr199, and Zinc coordination
**6**	−1.662	Zinc coordination
**7**	−4.881	Zinc coordination
**8**	−3.947	Thr199, Thr200, and Zinc coordination
**9**	−3.085	Asn62, Thr199, and Zinc coordination

**Table 4:** Predicted ADME properties for identified bioactive BERM-derived metabolites using SwissADME and QikProp tools.

Compound/Metabolite	Molecular Weight (g/mol)		Log Po/w		Log S		BBB Permeant		GI Absorption		Rule of Five (ROF)
	SWISS ADME	QikProp	SWISS ADME	QikProp	SWISS ADME	QikProp	SWISS ADME	QikProp	SWISS ADME	QikProp (%)	SwissADME
**Silymarin**	482.44	482.443	1.71	1.855	−4.50 Moderately soluble	−5.354	No	−2	Low	63.029	Yes; 0 violation
**1**	256.42	256.428	5.55	5.271	−5.31 Moderately soluble	−5.436	Yes	−2	High	88.223	Yes; 1 violation: MLOGP > 4.15
**2**	96.13	96.129	1.30	0.737	−0.88 soluble	−0.055	Yes	1	High	92.904	Yes; 0 violation
**3**	426.72	426.724	8.02	7.025	−6.74 Poorly soluble	−7.801	No	1	Low	100	Yes; 1 violation: MLOGP > 4.15
**4**	282.46	282.465	6.11	6.003	−5.39 Moderately soluble	−6.587	No	−2	High	91.435	Yes; 1 violation: MLOGP > 4.15
**5**	268.26	268.268	3.17	2.682	−5.68 Moderately soluble	−3.874	Yes	0	High	100	Yes; 0 violation
**6**	324.54	324.546	6.98	7.307	−6.51 Poorly soluble	−8.374	No	−1	Low	100	Yes; 1 violation: MLOGP > 4.15
**7**	222.24	222.243	3.46	3.55	−6.13 Poorly soluble	−3.521	Yes	1	High	100	Yes; 0 violation
**8**	194.18	194.184	−2.72	−1.918	1.74 Soluble	−0.337	No	−2	Low	50.691	Yes; 0 violation
**9**	436.62	436.631	3.93	3.809	−3.39 soluble	−5.759	No	−2	High	95.213	Yes; 0 violation

**Table 5:** The CYP enzymes inhibition profile for identified bioactive BERM-derived metabolites using the SwissADME web server.

Compound/Metabolite	CYP1A2	CYP2C19	CYP2C9	CYP2D6	CYP3A4
**Silymarin**	No	No	No	No	Yes
**1**	Yes	No	Yes	No	No
**2**	No	No	No	No	No
**3**	No	No	No	No	No
**4**	Yes	No	Yes	No	No
**5**	Yes	Yes	No	Yes	Yes
**6**	Yes	No	No	No	No
**7**	Yes	Yes	No	No	No
**8**	No	No	No	No	No
**9**	No	No	No	No	No

**Table 6:** Organ and endpoint toxicity predicted using the ProTox-II web server.

Compound Name	Classification					
	Organ Toxicity (% Probability)	Toxicity Endpoint (% Probability)				Oral Toxicity Prediction (Predicted Toxicity Class)
	Hepatotoxicity	Carcinogenicity	Immunotoxicity	Mutagenicity	Cytotoxicity	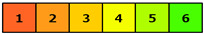
**Silymarin**	0.78 (inactive)	0.72 (inactive)	0.97 (active)	0.69 (inactive)	0.77 (inactive)	Class IV
**1**	0.52 (inactive)	0.63 (inactive)	0.99 (inactive)	1.0 (inactive)	0.74 (inactive)	Class IV
**2**	0.71 (inactive)	0.75 (inactive)	0.98 (inactive)	0.92 (inactive)	0.75 (inactive)	Class III
**3**	0.91 (inactive)	0.63 (inactive)	0.57 (active)	0.95 (inactive)	0.97 (inactive)	Class IV
**4**	0.55 (inactive)	0.64 (inactive)	0.99 (inactive)	1.0 (inactive)	0.71 (inactive)	Class II
**5**	0.71 (inactive)	0.53 (inactive)	0.72 (inactive)	0.68 (inactive)	0.95 (inactive)	Class V
**6**	0.59 (inactive)	0.61 (inactive)	0.91 (inactive)	0.97 (inactive)	0.59 (inactive)	Class V
**7**	0.70 (inactive)	0.69 (active)	0.99 (inactive)	0.54 (inactive)	0.75 (active)	Class V
**8**	0.96 (inactive)	0.83 (inactive)	0.99 (inactive)	0.68 (inactive)	0.84 (inactive)	Class VI
**9**	0.60 (inactive)	0.75 (inactive)	0.57 (active)	0.73 (inactive)	0.75 (inactive)	Class V

Class I: fatal if swallowed (LD_50_ ≤ 5), Class II: fatal if swallowed (5 < LD_50_ ≤ 50), Class III: toxic if swallowed (50 < LD_50_ ≤ 300), Class IV: harmful if swallowed (300 < LD_50_ ≤ 2000), Class V: may be harmful if swallowed (2000 < LD_50_ ≤ 5000), Class VI: non-toxic (LD_50_ > 5000).

## Data Availability

The datasets analyzed during the current study are available from the corresponding author on reasonable request.

## Supplementary Materials

The following supporting information can be downloaded at: https://www.mdpi.com/article/10.3390/metabo12111021/s1, Supplementary file S1. Certificate analysis of the mice diet.
