# Supplementary material for: Rhizophora mucronata Lam. (Mangrove) Bark Extract Reduces Ethanol-Induced Liver Cell Death and Oxidative Stress in Swiss Albino Mice: In Vivo and In Silico Studies
Source: Metabolites. 2022 Oct 25;12(11):1021. doi: 10.3390/metabo12111021 (PMC9698744; doi:10.3390/metabo12111021)
Supplement: Supplementary file 1 [file metabolites-12-01021-s001.zip › metabolites-1904448-supplementary.pdf]

Krishna Valley Agrotech LLP
Krishna Valley Agrotech LLP
An ISO 9001:2015 & GMP Certified Company
Corporate Office: Bungalow No. 5, Acacia Garden 1, Magarpatta City,Pune 411028,
Customer Care No: 120-61096718, Website: www.kvat.co.in
Factory address: E-43 & E3/14, MIDC, Kupwad block, Sangli, Maharashtra-416436

AF-1000M Rat &Mice Diets (Autoclavable)

CERTIFICATE OF ANALYSIS

Lot No : 223

Date of manufacture: 08.04.2022

Expiry date: 07.10.2022

Report date: 09.04.2022

KRISHNA VALLEY AGROTECH LLP

Authorized signatory

Proximate analysis

Analysis	Result %
Crude Protein	17.45
Ether extract	4.16
Crude Fiber	4.08
Moisture	8.95
Calcium	0.88
Phosphorus	0.67
Total ash	8.92
Gross Energy	3.0 Kcal/gm

Consolidated results obtained from one or more independent testing laboratories.

Analysis	Result	Units	Established Maximum concentration
**Heavy Metals**			
Arsenic	0.15	ppm	1.00
Cadmium	0.05	ppm	0.50
Lead	0.10	ppm	1.50
Mercury	0.02	ppm	0.20
**Mycotoxins**			
**Aflatoxin B1,B2, G1, G2**	<5.00	ppb	5.00
**Chlorinated Hydrocarbons**	<0.01	ppm	0.05
**Organophosphates**	<0.10	ppm	0.5
Phytoestrogen	Complies	µg/g	12
**Microbial Examination**			
Total Aerobic Count	Complies	CFU/gm	<1x10^3 CFU/gm
Mold Count	Absent	CFU/gm	Absent/10 gm
**Escherichia coli**	Absent	CFU/gm	Absent/10 gm
Salmonella	Absent	CFU/gm	Absent/10 gm
**Shigella**	Absent	CFU/gm	Absent/10 gm
Pseudomonas aeruginosa	Absent	CFU/gm	Absent/10 gm
